# Terahertz-Sensitive Diagnosis of Charge Carriers for Thermoelectrics

**DOI:** 10.34133/research.0724

**Published:** 2025-05-20

**Authors:** Ziran Zhao

**Affiliations:** Key Laboratory of Particle & Radiation Imaging (Ministry of Education), Department of Engineering Physics, Tsinghua University, Beijing 100084, China.

## Abstract

This commentary highlights the importance and implications of the study “Quadruple-band synglisis enables high thermoelectric efficiency in earth-abundant tin sulfide crystals”, led by C. Chang and L. Zhao, published in *Science*. They improved the thermoelectric efficiency by activating quadruple-band synglisis and facilitating carrier transport in tin sulfide crystals, and successfully developed an optical pump–terahertz probe technique in reflection mode to diagnose the carrier dynamics for high-conductivity bulk thermoelectric materials. This study inspires mutual promotion and complementary development between the fields of thermoelectrics and terahertz technology.

Advanced thermoelectric materials are highly desirable for thermoelectric technology to promote low-carbon and clean-energy utilization. Strategies to improve the energy conversion efficiency of thermoelectric materials involve decoupling the inherent conflicts between electrical and thermal transport, namely, optimizing electrical transport while minimizing thermal conductivity, including band structure engineering to boost power factor through doping/alloying, and nanostructuring to scatter phonons and reduce the lattice thermal conductivity [1,2]. In these synergistic approaches, characterizing carrier transport is a key task to assess the thermoelectric performance. The dynamic properties of charge carriers determine the basic electrical and thermal characteristics of thermoelectric materials. There exist various methods to diagnose charge carriers in emerging semiconductor materials, including Hall effect measurements, time-of-flight measurements, time-resolved optical and microwave techniques, transient photovoltage, and impedance spectroscopy. Among them, Hall effect measurements have been widely used for determining the electronic properties [3]. However, they have several main limitations, such as very limited measurement region for charge carriers, inaccurate measurement due to nonideal ohmic contact impedance, and only looking at steady-state carrier transport. Time-resolved optical techniques, particularly pulsed terahertz (THz) spectroscopy, have developed into powerful tools to reveal the ultrafast dynamic behavior of charge carriers in semiconductors [4].

Locating at the spectral regime of characteristic fingerprints of molecules [5–7], THz waves will interact specifically with physical, chemical, and biological systems that have the energetic transitions in meV range and picosecond-level characteristic lifetimes [8]. The ultrashort coherent THz pulses generated by femtosecond laser excitation enable optical pump–THz probe (OPTP) measurements for dynamic diagnosis of charge carriers with subpicosecond time resolution, as shown in Figure A. Nevertheless, it is extremely difficult to characterize the high-conductivity bulk samples, exactly the case of thermoelectric materials, by using the transmission-mode OPTP technique due to very limited penetration depth for THz waves.

**Figure.**
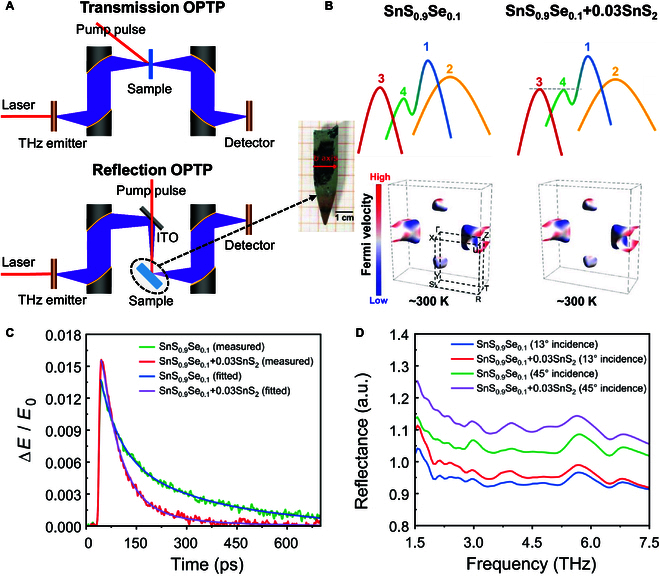
Schematic diagram of OPTP spectroscopy and its characterization results for SnS crystals. (A) Schematic illustration of transmission and reflection OPTP. (B) Schematic of the band structures and the corresponding Fermi surfaces for SnS_0.9_Se_0.1_ and SnS_0.9_Se_0.1_ + 0.03SnS_2_. The colored numbers and peaks indicate valence band maxima. (C) ROPTP measurements of SnS_0.9_Se_0.1_ and SnS_0.9_Se_0.1_ + 0.03SnS_2_ crystals. (D) Steady-state THz reflection spectra of the crystals. Reprinted with permission from Liu et al. [9]. Copyright 2025, American Association for the Advancement of Science.

Recently, C. Chang, L. Zhao, and collaborators from the National Innovation Institute of Technology and Beihang University presented to use reflection-mode OPTP (ROPTP) spectroscopy to study thermoelectric materials, in which the dynamics of charge carrier performance was successfully measured for high-conductivity bulk materials, published in *Science* [9]. This study presents a novel ROPTP spectroscopy system developed to address critical limitations in existing methodologies for investigating carrier dynamics. While recent advances in THz reflection spectroscopy have demonstrated surface conductivity characterization capabilities, conventional static non-pump-tunable configurations fundamentally restrict dynamic carrier behavior analysis. Furthermore, traditional transmission-mode measurements impose substantial constraints when probing bulk materials or highly conductive specimens. This angle-resolved ROPTP system uniquely enables simultaneous investigation of ultrafast carrier dynamics and their angular dependence—a critical parameter that has remained largely unexplored in previous reports. This configuration not only overcomes the inherent limitations of transmission geometry but also provides unprecedented angular resolution for probing anisotropic carrier transport phenomena in advanced functional materials. The demonstrated methodology represents a substantial advancement in noncontact, time-resolved characterization of photoinduced carrier behavior at material interfaces. In this study, to improve the thermoelectric efficiency in high-earth-abundance and low-cost SnS crystals, the authors first refined the composition of p-type Se-alloyed SnS crystal to obtain an optimized SnS_0.9_Se_0.1_ matrix, then introduced SnS_2_ to create more Sn vacancies in the SnS_0.9_Se_0.1_ matrix, and obtained SnS_0.9_Se_0.1_ + 0.03SnS_2_ (Figure B). Such innovative strategy promotes the convergence of energy and momentum of four valance bands so as to activate quadruple-band synglisis to enhance the carrier transport. To verify how the introduction of SnS_2_ facilitates the electrical transport performance, the authors developed an ROPTP system and conducted time-resolved THz spectroscopy measurements, as shown in Figure C. Based on the diagnosis, the slow decay constants of SnS_0.9_Se_0.1_ and SnS_0.9_Se_0.1_ + 0.03SnS_2_ are extracted as 300.3 and 195.2 ps, respectively, demonstrating the evidence of high carrier mobility after introducing SnS_2_ into SnS_0.9_Se_0.1_. By conducting the steady-state THz measurements under vacuum condition, the reflection waves of SnS_0.9_Se_0.1_ + 0.03SnS_2_ are higher than that of SnS_0.9_Se_0.1_ in both high-angle-incident and low-angle-incident conditions (Figure D), illustrating the enlarged carrier concentration [9]. Note that the sensitive diagnosis of charge carrier dynamic process for the high-conductivity bulk thermoelectric materials benefits from the invention of ROPTP technique and its nondestructive, noncontact, and subpicosecond time-resolved capabilities. First, OPTP measurements can reveal the dynamic process in a very high time resolution on order of subpicoseconds, enabling real-time tracking of ultrafast carrier dynamic processes such as excitation, recombination, and energy relaxation of carriers. In Figure C, the carrier relaxation time can be easily obtained by this technique. Besides, THz waves have low photon energy and are highly sensitive to changes in carrier concentration and distribution in semiconductors. They can noninvasively detect the optoelectronic properties of materials, avoiding damage to samples by high-energy photons. Also, by THz transmission or reflection measurements, dynamic information of charge carriers can be obtained without direct contact with the sample, making it suitable for material research with fragile or complex structures.

This study opens an exciting perspective toward the application of THz technology in thermoelectrics. Actually, in return, thermoelectric materials also exhibit promising potential for detection of THz waves via photothermoelectric (PTE) effect. In 2014, our group first discovered the PTE response of carbon nanotube–metal heterostructures under THz wave excitation [10]. Since then, this new photothermal detection mechanism that can break through photon energy limitations and be sensitive to the entire THz range was proposed and developed. Starting from the two key physical processes of hot-carrier excitation and transport, we have constructed a comprehensive theoretical framework of THz PTE detectors and presented a series of approaches to optimize the detection performance [11]. Compared with other photothermal THz detectors, PTE detectors hold the feature of ultrabroadband response without bandgap limitation and unique advantages of ultrafast response, zero-bias operation, low power consumption, and simple device geometry, making them particularly suitable for detection of medium-high frequency THz waves (e.g., >3 THz) and development of multifunctional, compact, and low-cost on-chip sensors [12]. In PTE effect, the conversion efficiencies of two stages, photo-to-thermal and thermal-to-electric, determine the detection sensitivity. This means that the criteria for good THz PTE detection candidate materials are high photothermal absorption to THz waves and superior thermoelectric figure of merit (ZT, depending on the performance of thermoelectric materials). Consequently, materials with high THz absorption and good thermoelectric performance have been pursued for serving as the photoactive channel of THz PTE detectors. Certainly, there is a compromise between detection sensitivity and bandwidth if using thermoelectric materials for THz PTE detection. High thermal-to-electric efficiency favors a material with high conductivity and long momentum scattering time, which leads to THz absorption primarily at lower frequencies, or limited photo-to-thermal efficiency. Combining thermoelectric materials with a THz absorber would be a potential solution to achieve highly sensitive PTE detection within an ultrabroadband spectral range. In general, inspired by this study, not only THz technology can diagnose the carrier dynamics of earth-abundant tin sulfide crystals with quadruple-band synglisis but also such materials would be expected to act as good candidates for THz PTE detection.

In addition, we recognize that the characteristic frequencies of lattice phonon oscillations predominantly reside within the THz spectral regime. This physical correspondence suggests that THz dynamical traces may not only encode fundamental carrier dynamics but also potentially manifest lattice cooling signatures—a synergistic phenomenon that has been recognized as particularly intriguing. Such dual-probing capability presents intriguing opportunities for exploring coupled electron–phonon interactions, with potential implications for guiding rational design strategies in advanced functional materials through systematic elucidation of energy relaxation pathways.

Overall, THz radiation with low photon energy provides a nondestructive solution to probe free carrier concentration and mobility via the photo-induced change in the surface conductivity while being insensitive to the excitons and trapped carriers. It can be anticipated that THz technology will contribute to the semiconductor design and in situ characterization diagnosis, avoiding any artifacts due to the electronic contacts to the mesoporous material.
